# Florid Lobular Carcinoma In Situ (F-LCIS) Showing Predominantly Hypoechoic Masses With Obscured Margins and No Attenuated Posterior Echoes on Ultrasound: A Case Report

**DOI:** 10.7759/cureus.110415

**Published:** 2026-06-07

**Authors:** Kasumi Arizumi, Shoji Oura

**Affiliations:** 1 Department of Surgery, Kishiwada Tokushukai Hospital, Kishiwada, JPN

**Keywords:** florid type lobular carcinoma in situ, no attenuated posterior echoes, obscured margins, predominant internal low echoes, small masses

## Abstract

Florid lobular carcinoma in situ (F-LCIS) is a rare disorder and has no established image findings to date. A 63-year-old woman was referred to our hospital for the detailed examination of the abnormality on screening mammography. Mammography showed a focal asymmetric density in the left breast. Ultrasound showed multiple round/oval masses with obscured margins, predominant low internal echoes, and no attenuated posterior echoes. MRI of the masses showed low signals on T1-weighted images, high signals on fat-suppressed T2-weighted images, fast enhancement followed by a washout pattern, and presumed ductal spread to the nipple base on dynamic studies. After the pathological diagnosis of predominant LCIS on core needle biopsy, the patient underwent nipple-sparing mastectomy and sentinel node biopsy followed by immediate breast reconstruction using the extended latissimus dorsi musculocutaneous flap. Postoperative pathological study showed that all four masses, except for the largest one, were F-LCIS, and the largest mass, 9 mm in pathological size, was predominant florid ductal carcinoma in situ (F-DCIS) with invasive components. The patient recovered uneventfully, was discharged on the 13th day after surgery, has been well for four months on adjuvant letrozole therapy, and is scheduled for long-term follow-up on an outpatient basis. Diagnostic physicians should note that predominantly hypoechoic masses with obscured margins and no attenuated posterior echoes may be important ultrasound findings of F-LCIS.

## Introduction

Breast surgeons generally treat ductal carcinoma in situ (DCIS) with some kind of surgery [1]. Breast surgeons, however, regard the vast majority of lobular carcinoma in situ (LCIS) only as a risk factor for invasive breast cancer and do not treat it surgically [2,3]. LCIS has three subtypes: classic, pleomorphic, and florid subtypes, which have different biologies. For example, classic LCIS has extremely indolent characteristics, but the other two subtypes often have invasive components. Many researchers, therefore, recommend that pleomorphic LCIS and florid LCIS (F-LCIS) be treated with some kind of surgery, similar to DCIS [4-6].

LCIS has no E-cadherin expression, neither in invasive lobular carcinoma (ILC), and frequently has two or three LCIS subtype components. ILC accounts for 5-10% of all breast cancers and has a better prognosis than invasive ductal carcinoma [7]. ILC has characteristic image findings due to the presence of abundant fibrous components, which resemble those of scirrhous-type invasive ductal carcinoma. Abundant fibrous components in ILC make obscured mass margins, distortion of the mammary gland, and attenuated posterior echoes [8]. In contrast, LCIS has much fewer fibrous components than ILC and therefore has variable imaging appearances, leading to incomplete image characterization to date. Some studies have evaluated LICS about the pathological and mammographic findings, while few studies have evaluated LCIS about ultrasound findings to date [6].

We herein report F-LCIS showing characteristic ultrasound findings closely correlated with pathological findings.

## Case presentation

A 63-year-old woman with a history of malignant melanoma was referred to our hospital for the detailed examination of the abnormality on screening mammography. She had no family history of breast or ovarian cancer, but there was a paternal history of colon cancer. Mammography showed focal asymmetric density (FAD) and no calcifications suggestive of malignancy in the left breast (Figure 1).

**Figure 1:**
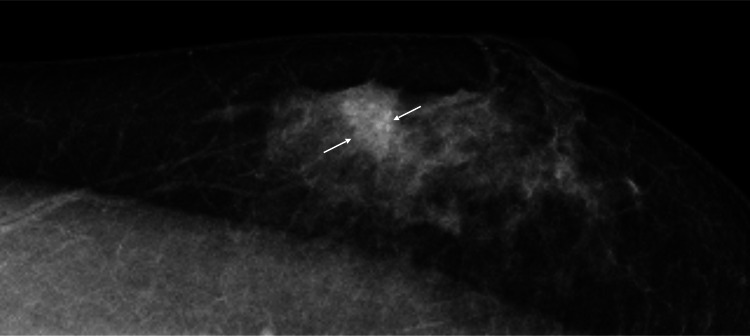
Mammography findings Mammography showed focal asymmetric density (arrows) in the left breast.

The patient had neither any clearly defined masses nor axillary lymph node swelling in the left breast, but had an induration in the areas consistent with FAD. Ultrasound showed multiple round/oval masses, up to 16 mm in size, with obscured margins, predominant internal low echoes, no attenuated posterior echoes, and no regional lymph node swelling (Figure 2).

**Figure 2:**
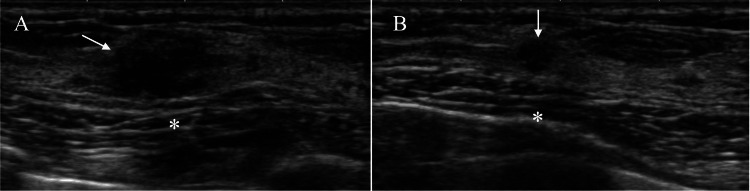
Ultrasound findings Ultrasound showed multiple oval (A) or round (B) masses (arrow) with low internal echoes accompanied by focal internal punctate high echoes and no attenuated posterior echos (asterisk).

Magnetic resonance imaging (MRI) of the breast masses showed low signals on T1-weighted images, high signals on fat-suppressed T2-weighted images, and fast enhancement followed by a washout pattern and presumed ductal spread to the nipple base on dynamic studies (Figure 3).

**Figure 3:**
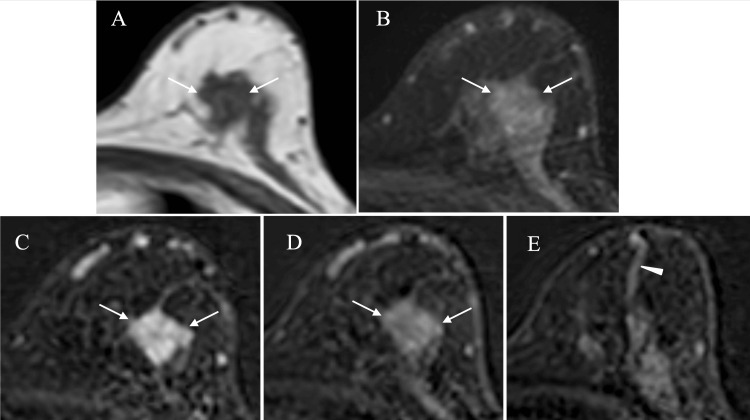
Magnetic resonance imaging (MRI) findings MRI of the largest mass (arrows) showed low signals on T1-weighted images (A) and weak high signals on fat-suppressed T2-weighted images (B). MRI further showed early enhancement (C) followed by a wash out pattern (D) and presumed ductal spread to the nipple base (E, arrowhead) on dynamic studies.

We, therefore, did a core needle biopsy on the largest mass under the diagnosis of breast cancer. Pathological study of the core needle biopsy specimen showed signet-ring cell carcinoma-like atypical cells growing mainly in the terminal duct lobular units with connective tissue proliferation and focal invasion in linear and isolated fashions, leading to the diagnosis of ILC. The patient, therefore, underwent nipple-sparing mastectomy and sentinel node biopsy followed by immediate breast reconstruction using the extended latissimus dorsi musculocutaneous flap. Frozen section showed no cancer metastasis to the sentinel node, and no cancer spread to the nipple base. Postoperative pathological study showed that four masses, with three being F-LCIS and the largest mass being predominantly F-DCIS, 9 mm in size, with invasive components within it (Figure 4).

**Figure 4:**
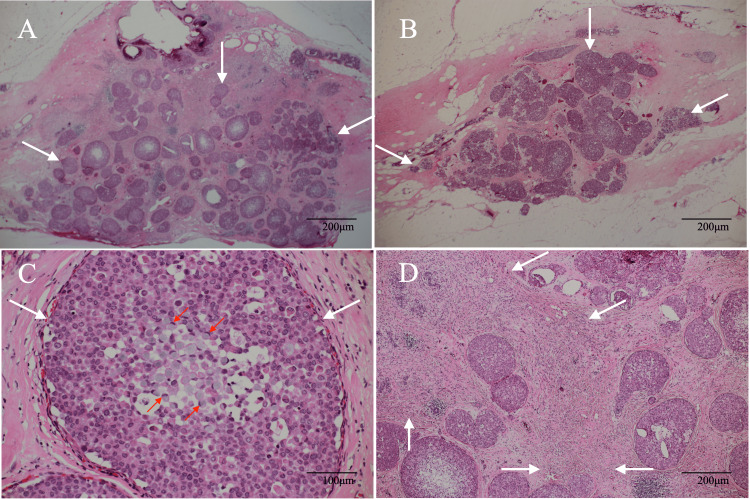
Pathological findings Low-magnified view (H&E, ×40) showed (A) the largest mass, 9 mm in size, (white arrows) of multiple florid LCIS foci and (B) a mass, 4 mm in size (white arrows), showing similar pathological findings to and located adjacent to the largest mass. Magnified view showed (C) marked dilatation of the terminal lobules due to the proliferation of cancer cells (white arrows) resembling to classic LCIS and showed some signet-ring cell-like cells (red arrows) (H&E, ×200) and (D) invasive lobular carcinoma cells (white arrows) among florid LCIS foci (H&E, x100) LCIS: lobular carcinoma in situ

The patient recovered uneventfully, was discharged on the 13th day after the operation, has been well for four months on adjuvant letrozole therapy, and is scheduled for long-term follow-up on an outpatient basis.

## Discussion

The patient had small LCIS foci and the largest tumor, 9 mm in pathological size, with predominant LCIS and ILC components. Ultrasound showed that LCIS components had basically low internal echoes and focal punctate high echoes. Pathological study clarified that solid cancer clusters had many signet-ring cell carcinoma-like cancer cells. Different acoustic impedance between the mucus parts of the signet ring cell-like cancer cells and the non-signet ring cell-like cancer cells presumably made the ultrasound waves be backscattered, leading to the formation of internal punctate high echoes [8]. Solid foci of the F-LCIS, however, had aggregated to form the tumor, leading to the generation of the predominant internal low echoes in this case.

This breast cancer had fibrous components around both the LCIS and ILC foci, but the amount of them was much less than that generally observed in ILC. In addition, small sizes of each LCIS foci had presumably made the ultrasound wave attenuation much less than that of ILC. It is well known that such atypical ultrasound findings, i.e., no remarkable posterior echo attenuation despite the presence of fibrous components in masses, are also often observed in small scirrhous-type invasive ductal carcinomas with abundant fibrous components [9]. No attenuated posterior echoes, therefore, may be an important ultrasound finding of F-LCIS.

Notably, all LCIS foci had indistinct mass margins in this case. This finding is well explained by the following two mechanisms. First, all LCIS foci located in the lobules were pathologically encompassed by the surrounding fibrous stroma and presumably had similar acoustic impedance to the fibrous stroma. Second, the tumor, composed of multiple small LCIS foci, had no large interfaces that would elicit ultrasound wave reflection. No ultrasound reflection, therefore, occurred at the tumor margins, which generated the obscured mass margins [10]. Obscured mass margins, therefore, should be another important ultrasound finding of F-LCIS.

MRI of the F-LCIS foci showed low signals on T1-weighted images, weak high signals on fat-suppressed T2-weighted images, and fast enhancement and late very weak enhancement on dynamic studies. These MRI findings could be common to both the florid type and pleomorphic LCISs when taking into consideration both the aggregation of LCIS foci of certain sizes and the surrounding fibrous stroma.

It is well known that differentiation between pleomorphic LCIS and F-LCIS is often difficult even on pathological evaluation. Difficult pathological discrimination naturally leads to the difficult image differentiation of these two disorders. F-LCIS, however, is defined that acini of it has the diameter at least larger than that equivalent to 40-50 cancer cells, i.e., markedly distended acini. In addition, F-LCIS has less intervening fibrous stroma than pleomorphic LCIS [11]. Multiple oval/round masses with low internal echoes and no attenuated posterior echoes, therefore, may favor the diagnosis of F-LCIS over that of pleomorphic LCIS.

## Conclusions

Breast specialists rarely encounter mass-forming LCIS lesions. Among them, differentiation between pleomorphic LCIS and F-LCIS is often difficult even by pathological evaluation. Larger acini in F-LCIS make us favor the diagnosis of F-LCIS over pleomorphic LCIS when detecting multiple small masses. Diagnostic physicians, therefore, should note that a mass-forming LCIS may be F-LCIS when having oval/round small hypoechoic masses with obscured margins and no attenuated posterior echoes. It, however, is necessary to analyze the correlation between the pathological features and imaging of f-LCIS in a large number of cases in the future.

## References

[1] Ryser MD, Thomas SM, Li Y (2025). Cancer outcomes in women without upfront surgery for ductal carcinoma in situ: observational cohort study. BMJ.

[2] Morrow M, Schnitt SJ, Norton L (2015). Current management of lesions associated with an increased risk of breast cancer. Nat Rev Clin Oncol.

[3] Bowman K, Munoz A, Mahvi DM, Breslin TM (2007). Lobular neoplasia diagnosed at core biopsy does not mandate surgical excision. J Surg Res.

[4] Middleton LP, Palacios DM, Bryant BR, Krebs P, Otis CN, Merino MJ (2000). Pleomorphic lobular carcinoma: morphology, immunohistochemistry, and molecular analysis. Am J Surg Pathol.

[5] Singh K, Sung CJ, Quddus MR (2020). Descriptive study comparing outcomes of classic and nonclassic lobular carcinoma in situ (florid and pleomorphic) initially diagnosed on core needle biopsy. Breast J.

[6] Singh K, Paquette C, Kalife ET, Wang Y, Mangray S, Quddus MR, Steinhoff MM (2018). Evaluating agreement, histological features, and relevance of separating pleomorphic and florid lobular carcinoma in situ subtypes. Hum Pathol.

[7] Li CI, Moe RE, Daling JR (2003). Risk of mortality by histologic type of breast cancer among women aged 50 to 79 years. Arch Intern Med.

[8] Kitano Y, Oura S, Honda M (2025). Accurate pathological prediction of small breast cancer with pathological component-based image evaluation: a case report. Cureus.

[9] Takano Y, Oura S, Honda M (2025). Image findings of breast cancer-related elastosis: a case report. Case Rep Oncol.

[10] Takaoka K, Oura S (2025). A case of breast cancer arising from a florid adenosis nodule. Cureus.

[11] Chen YY, Decker T, KIng TA, Palacios J, Reis-Filho JS, Shin SJ, Simpson PT Lobular carcinoma in situ. WHO Classification of Tumours: Breast Tumours.

